# Supplementary material for: Constraint-Based, Score-Based and Hybrid Algorithms to Construct Bayesian Gene Networks in the Bovine Transcriptome
Source: Animals (Basel). 2022 May 19;12(10):1305. doi: 10.3390/ani12101305 (PMC9138007; doi:10.3390/ani12101305)
Supplement: Supplementary file 1 [file animals-12-01305-s001.zip › animals-1670560-supplementary.pdf]

Constraint-Based, Score-Based and Hybrid Algorithms to Construct Bayesian Gene Networks in the Bovine Transcriptome

Supplementary Table S1: A literature review on bovine transcriptomic modeling using other methodologies

Study	Tissue	Trait	Model	Data	Results
Wilkinson et al. 2012	Peripheral blood cells	Disease susceptibility	LIMMA, IPA, DAVID	DNA microarray	IL7R, JUN, TNFRSF90, and ZAP70
Ghaderi-Zefrehei et al. 2015	Immune system	Immune system	SMILE, GeNIe	RT-PCR	GM-CSF, IL-2, IFN-γ, IL-6, IL-8, and TNF-α
Fortes et al. 2012	Hypothalamus	Body weight, hip height, average daily gain, back fat thickness, intramuscular fat	GWAS associated weighted matrix	GWAS	978 Genes, 1,555 important SNP, TF'S (ZMAT3, STAT6, RFX4, PLAGL1, and NR6A1)
Moran et al. 2017 (Ghaderi-Zefrehei et al., 2015)	Endometrium	Fertility	Gephi	RNA Seq	123 genes from three physiologically relevant networks (1) actin and cytoskeletal components; (2) immune function; and (3) ion transportation, 403 DE genes
Ramayo-Caldas et al. 2014 (Gray et al., 2006)	Intramuscular fat	Intramuscular fat	Associated weight matrix	SNP	Three TF (HNF4G, PARAGC1A, FOXP3); Genes (CAPN6, STC2, MAP2K4, EYA1, COPS5, XKR4, NR2E1, TOX, ATF1, ASPH, TGS1, and TTPA)
Alexandre et al. 2015	Liver	Feed efficiency	(WGCNA) R-package	RNA-Seq	Genes (SOD3, FASN, GADD45G, CYP2E1, ENSBTAG00000038430, mir-2904-3, RHOB, NR0B2) 8 DE, 34 Module, 463 differentially co-expressed genes, 8 key regulators

Cánovas et al. 2014	Hypothalamus, pituitary, endometrium, ovary, uterus	Puberty	Partial correlation and information theory (PCIT) algorithms	GWAS, RNA-Seq	5 TF's (PROP1, DACH2, FOXA1, PITX2, SIX6)
Gonçalves et al. 2018	Skeletal muscle	Beef tenderness	Partial correlation with information theory (PCIT), phenotypic impact factor (PIF) and the regulatory impact factor (RIF)	RNA-Seq	Genes (USP2, GBR10, ANO1, TMBIM4, MB, ENO3, CA3); MicroRNA (bta-mir-133a-2 and bta-mir-22)
Neupane et al., 2017	Endometrium	Pregnancy	GWAS	SNP	TNF, TP53, SOX2, and OCT4

Abbreviations: LIMMA: Linear Models for Microarray Data ; IPA: Ingenuity Pathway Analysis; DAVID: Database for Annotation, Visualization and Integrated Discovery IL7R: ; JUN: ; TNFRSF90: ; ZAP70: ; SMILE: ; RT-PCR: ; GM-CSF: ; IL-2: interleukin-2; IFN-γ: interferon γ; TNF-α: tumor necrosis factor α; GWAS: ; SNP: single nucleotide polymorphism; Transferrin (TF): ; Zinc Finger Matrin-Type 3 (ZMAT3): ; Signal Transducer And Activator Of Transcription 6 (STAT6): ; Regulatory Factor X4 (RFX4): ; PLAG1 Like Zinc Finger 1 (PLAGL1): ; Nuclear Receptor Subfamily 6 Group A Member 1 (NR6A1): ; DE: differentially expressed?; Hepatocyte Nuclear Factor 4 Gamma (HNF4G): ; PPARG coactivator 1 alpha (PARAGC1A): ; Forkhead Box P3 (FOXP3): ; Calpain 6 (CAPN6): ; Stanniocalcin 2 (STC2): ; Mitogen-Activated Protein Kinase Kinase 4 (MAP2K4): ; EYA Transcriptional Coactivator And Phosphatase 1 (EYA1): ; COP9 Signalosome Subunit 5 (COPS5): ; XK Related 4 (XKR4): ; Nuclear Receptor Subfamily 2 Group E Member 1 (NR2E1): ; Thymocyte Selection Associated High Mobility Group Box (TOX): ; Activating Transcription Factor 1 (ATF1): ; Aspartate Beta-Hydroxylase (ASPH): ; Trimethylguanosine Synthase 1 (TGS1): ; Alpha Tocopherol Transfer Protein (TTPA): ; Superoxide Dismutase 3 (SOD3): ; Fatty Acid Synthase (FASN): ; Growth Arrest And DNA Damage Inducible Gamma (GADD45G): ; Cytochrome P450 Family 2 Subfamily E Member 1 (CYP2E1): ;

Ras Homolog Family Member B (RHOB): ; Nuclear Receptor Subfamily 0 Group B Member 2 (NR0B2): ; PROP Paired-Like Homeobox 1 (PROP1): ; Dachshund Family Transcription Factor 2 (DACH2): ; Forkhead Box A1 (FOXA1):; Paired Like Homeodomain 2 (PITX2): ; SIX Homeobox 6 (SIX6): ; Ubiquitin Specific Peptidase 2 (USP2): ; Growth Factor Receptor-Bound Protein 10 (GBR10): ; Anoctamin 1 (ANO1): ; Transmembrane BAX Inhibitor Motif Containing 4 (TMBIM4): ; Myoglobin (MB): ; Enolase 3 (ENO3): ; Carbonic Anhydrase 3 (CA3): ; Tumor Necrosis Factor (TNF): ; Tumor Protein P53 (TP53): ; SRY-Box Transcription Factor 2 (SOX2): ; Octamer-Binding Transcription Factor 4 (OCT4).

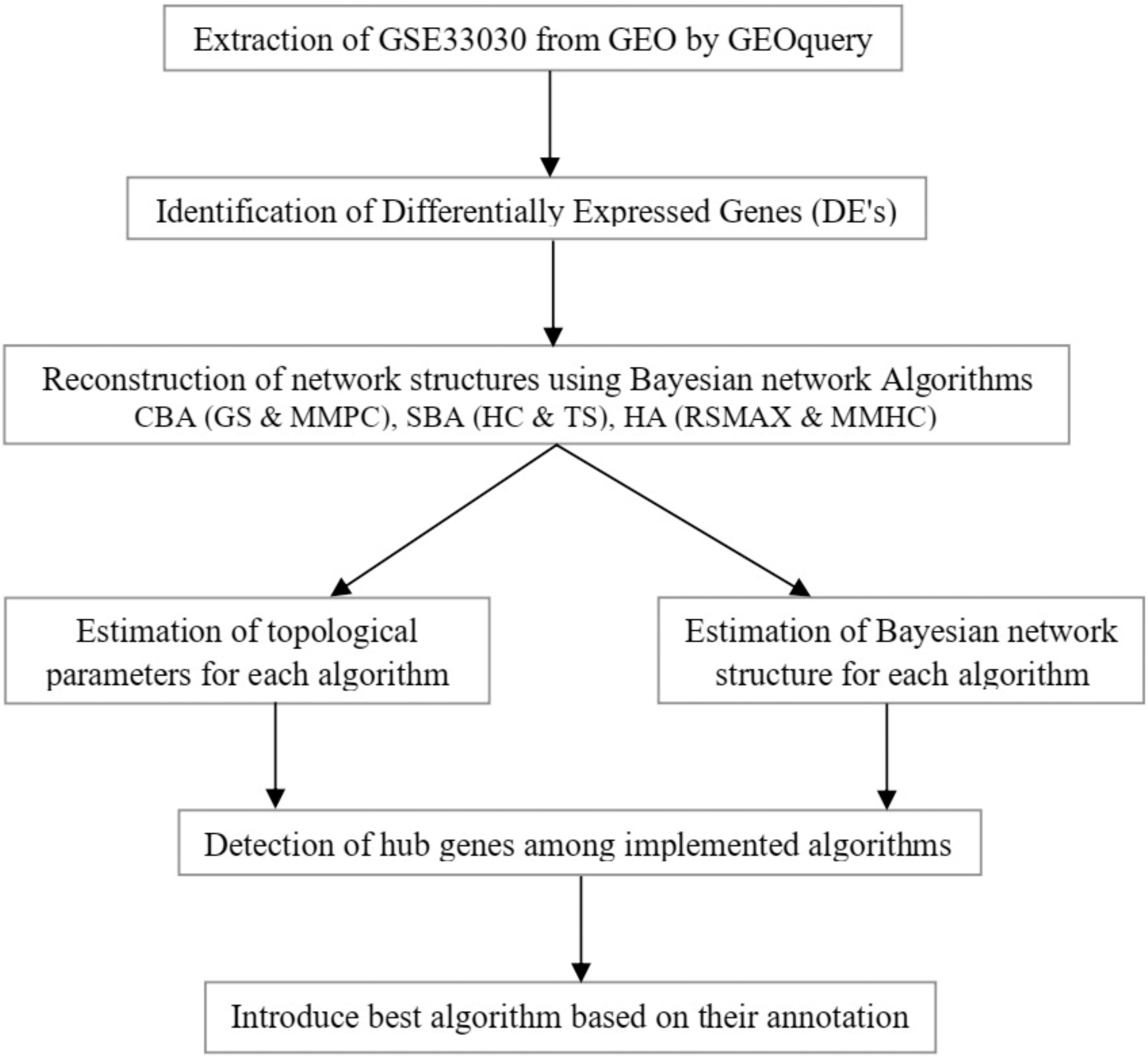

Supplementary Figure S1: Flow diagram of the proposed method. GEO - gene expression omnibus; CBA - constraint-based algorithms; SBA - score-based algorithms; HA - hybrid algorithms; GS - grow shrink; MMPC - Max-Min parent children; HC - hill climbing; TS - Tabu search; MMHC - Max-Min hill climbing; RSMAX - restricted maximum.

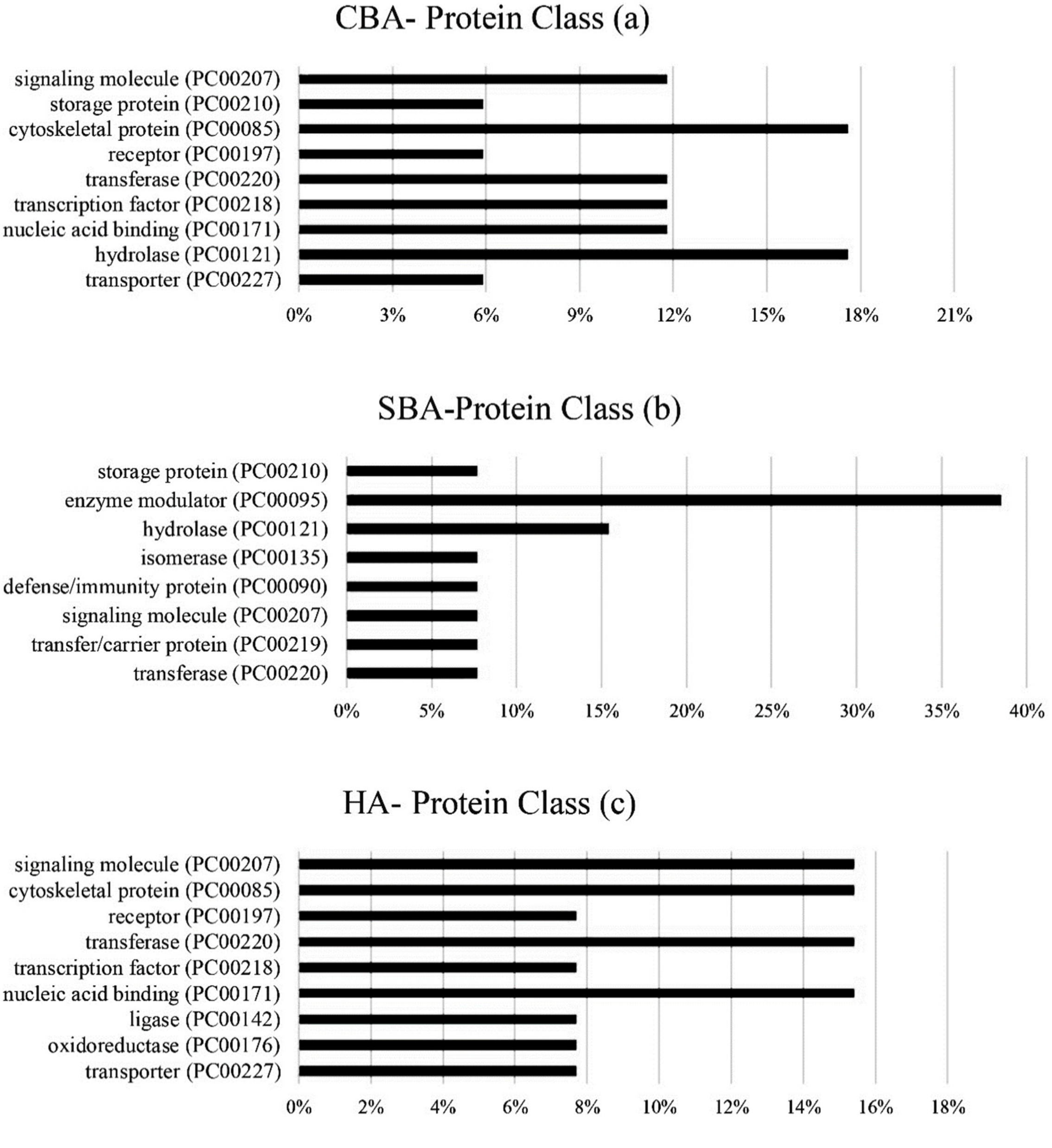

Supplementary Figure S2: Protein class of identified hub genes from (a) CBA, (b) SBA and (c) HA algorithms. CBA - constraint-based algorithms; SBA - score-based algorithms; HA - hybrid algorithms.
